# Naturally occurring 3*RS*, 7*R*, 11*R*-phytanic acid suppresses in vitro T-cell production of interferon-gamma

**DOI:** 10.1186/s12944-018-0793-6

**Published:** 2018-06-23

**Authors:** Tomonori Nakanishi, Ibuki Motoba, Mayuko Anraku, Ryoji Suzuki, Yuto Yamaguchi, Laurie Erickson, Nozomu Eto, Kazuhiro Sugamoto, Yohichi Matsushita, Satoshi Kawahara

**Affiliations:** 1 Department of Biochemistry and Applied Biosciences, Faculty of Agriculture, University of Miyazaki, 1-1 Gakuenkibanadai-nishi, Miyazaki, 889-2192 Japan; 2 Department of Applied Chemistry, Faculty of Engineering, University of Miyazaki, 1-1 Gakuenkibanadai-nishi, Miyazaki, 889-2192 Japan; 3 Department of Biology, Harold Washington City College of Chicago, 30 E. Lake St, Chicago, IL 60601 USA; 4 Department of Health Sciences, Blitstein Institute of Hebrew Theological College, 2606 W. Touhy Ave, Chicago, IL 60645 USA

**Keywords:** 3*RS*, 7*R*, 11*R*-phytanic aid, T-cell, IFN-γ production, NF-κB activity, PPARα

## Abstract

**Background:**

Among the eight stereoisomers of phytanic acid (PA), the 3*RS*, 7*R*, 11*R*-isomer is naturally occurring and is present in foods and the human body. PA is considered to have possible health benefits in the immune system. However, it remains undetermined whether these effects are elicited by the 3*RS*, 7*R*, 11*R*-PA isomer, because previous studies used a commercially available PA whose isomer configuration is unknown. In this study, we synthesized a preparation of 3*RS*, 7*R*, 11*R*-PA, and investigated its in vitro immunomodulatory effects, especially the T-cell production of interferon (IFN)-γ, which is associated with various autoimmune diseases. This study also investigated the effects of 3*RS*, 7*R*, 11*R*-PA on NF-κB activity in order to address the mechanism of its immunomodulatory effects.

**Methods:**

Mouse splenocytes and purified T-cells were stimulated with T-cell mitogens and incubated with 3*RS*, 7*R*, 11*R*-PA, followed by evaluation of IFN-γ production. The effect of 3*RS*, 7*R*, 11*R*-PA on NF-κB activity was also investigated using an A549 cell line with stable expression of an NF-κB-dependent luciferase reporter gene.

**Results:**

3*RS*, 7*R*, 11*R*-PA significantly reduced in vitro IFN-γ production at both the protein and mRNA levels, and was accompanied by decreased expression of T-bet, a key regulator of Th1 cell differentiation. The results indicated that NF-κB-mediated transcriptional activity was significantly decreased by 3*RS*, 7*R*, 11*R*-PA and that GW6471, an antagonist of peroxisome proliferator activated receptor α (PPARα), abrogated the inhibitory effect of 3*RS*, 7*R*, 11*R*-PA on NF-κB activity.

**Conclusions:**

The present study suggests that 3*RS*, 7*R*, 11*R*-PA is a functional and bioactive fatty acid, and has a potentially beneficial effect for amelioration of T-cell mediated autoimmune diseases. This study also indicates that interference in the NF-κB pathway via PPARα activation is a potential mechanism of the immunomodulatory effects of 3*RS*, 7*R*, 11*R*-PA.

**Electronic supplementary material:**

The online version of this article (10.1186/s12944-018-0793-6) contains supplementary material, which is available to authorized users.

## Background

Phytanic acid (3, 7, 11, 15-tetramethyl-hexadecanoic acid, PA) is a branched-chain fatty acid and originates from the phytol side chain of chlorophyll. Some microorganisms inhabiting the rumen of ruminants produce phytol from chlorophyll, after which PA is formed via oxidation of phytol to phytenic acid. Therefore, dairy products and ruminant meat as well as some marine lipids are rich sources of PA [1]. Because humans are not capable of producing phytol from chlorophyll, PA in the human body is exclusively derived from the above foods.

PA is a ligand for several subtypes of peroxisome proliferator activated receptor (PPAR) [2, 3]. PPARs regulate gene expression in cellular differentiation, development and metabolism, and are attractive molecular targets for human metabolic disease [4], type 2 diabetes [5] and autoimmune diseases [6]. PA has been shown to stimulate glucose uptake in primary porcine myotubes, suggesting a potential role of PA in the management of insulin resistance [7]. Our previous studies addressing the immunomodulatory effects of PA have demonstrated that PA inhibits T-cell production of cytokines, such as interferon (IFN)-γ, which are associated with various autoimmune diseases [8]. Based on these findings, PA is now recognized as a food component with possible benefits for human health, albeit the precise molecular mechanism still remains unclear [9].

PA has three chiral centers at carbon positions 3, 7 and 11, which means that there are theoretically eight stereoisomers. In nature, the methyl groups at 7 and 11 are in the R configuration because they are in chlorophyll-derived phytol (2*E*, 7*R*, 11*R*-isomer). The configuration at position 3 in PA can be *R* or *S*, and consequently naturally occurring PA (nPA) is the diastereomeric mixture of 3*R*, 7*R*, 11*R*- and 3*S*, 7*R*, 11*R*-isomers [10]. However, most previous studies have used commercially available PA which is a mixture of natural and non-natural isomers or whose isomer information is unknown. Therefore, there are unanswered questions of whether nPA can elicit the above beneficial effects. Because individual isomers of fatty acids differ widely in their biological effects [11], evaluation of nPA is important to determine the biochemical and physiological effects of PA.

Here, we synthesized a mixture containing equal amounts of the 3*R*, 7*R*, 11*R*- and the 3*S*, 7*R*, 11*R*-isomers in order to evaluate the in vitro immunomodulatory effects of nPA on IFN-γ production by T-cells, to address the potential for prevention of autoimmune disease. We also investigated the effects of nPA on NF-κB-mediated transcriptional activity to elucidate the mechanism by which nPA elicits the immunomodulatory effects.

## Methods

### Preparation of nPA from phytol

**Scheme 1:**
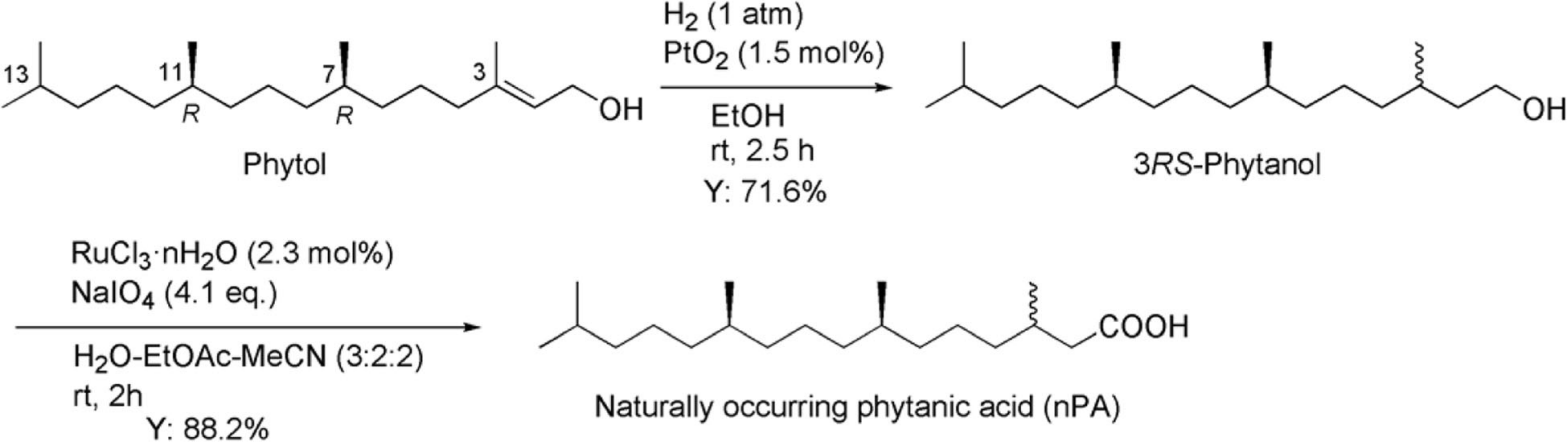
Conversion of phytol into naturally occurring phytanic acid (nPA)

Phytol 95 containing 92% of phytol derived from chlorophyll was purchased from Tama Biochemical CO., Ltd. (Tokyo, Japan) and was purified by column chromatography on silica gel with hexane−diethyl ether (2:1) as an eluent to give pure phytol. Phytol was converted into nPA (Scheme 1). Hydrogenation of phytol using Adams’ catalyst gave 3*RS*-phytanol according to Patton and Benson [12]. For conversion of phytanol into PA, chromic acid oxidation under acidic conditions has been almost exclusively used [12–14], but the use of noxious chromic acid should be avoided if possible. Therefore, we applied ruthenium tetroxide-catalyzed oxidation with NaIO_4_ [15] instead of the chromic acid oxidation.

Briefly, to a solution of phytol (5.05 g) in ethanol (170 mL) was added 58.6 mg of Adams’ catalyst (PtO_2_). After the atmosphere of reaction vessel had been replaced with hydrogen, the reaction mixture was stirred at room temperature for 2.5 h under 1 atm of hydrogen. The catalyst was removed from the mixture by filtering through a celite pad. The filtrate was evaporated on a rotary evaporator to 5.7 g of the crude product as a colorless oil. The crude product was purified by column chromatography on silica gel (50 g) with hexane and then hexane-ethyl acetate (20:1) as an eluent. Phytane (1.27 g) produced by hydrogenolysis was obtained in 26.4% yield from hexane-eluted fraction, and 3*RS*-phytanol (3.64 g) in 71.6% as a colorless oil from the fraction eluted with hexane-ethyl acetate (20:1). The spectral data for synthesized phytanol coincided with those reported by Burns et al. [14].

3*RS*-phytanol (3.58 g) was dissolved in a mixed solvent of acetonitrile (24 mL) and ethyl acetate (24 mL), and water (36 mL) was added to this solution. To the mixture were added sodium periodate (10.53 g) and ruthenium chloride hydrate (62.2 mg) sequentially. The reaction mixture was stirred at room temperature for 2 h. The reaction mixture was filtered to remove insoluble solids, and the organic layer was separated from the two-layered filtrate. The aqueous layer was extracted with ethyl acetate (2 × 60 mL). The combined organic layers were dried over anhydrous sodium sulfate, evaporated on a rotary evaporator, to afford 3.79 g of the crude product as a brownish yellow oil. Purification of the crude product on silica gel (70 g) with hexane−ethyl acetate−acetic acid (20:1:0.1) gave the desired nPA (3.03 g) as a colorless oil in 88.2% yield. Its spectral data coincided with those reported by Burns et al. [14].

In this study, nPA was used either as the form of free fatty acids or as nPA-bovine serum albumin (BSA) conjugates. The conjugation of nPA with BSA was carried out as previously described [16].

### Animals and cells

Female C57BL/6 J mice aged 6 weeks were obtained from Japan SLC, Inc. (Shizuoka, Japan). The spleens of mice aged between 8 to 12 weeks were aseptically collected and teased into single-cell suspensions. T-cells were purified from mouse splenocytes by negative immunomagnetic cell sorting (Pan T cell isolation kit II, Miltenyi Biotec, Bergisch Gladbach, Germany). Mouse splenocytes and purified T-cells were incubated in RPMI-1640 medium containing 10% foetal calf serum, 100 units/mL penicillin and 100 μg/mL streptomycin. Animals were used in accordance with the guidelines for the care and use of laboratory animals at the University of Miyazaki and Law No. 105 of the Japanese government. All experimental protocols were approved by the University of Miyazaki (approval number: 2014–002). A549 human lung epithelial cells with stable expression of an NF-κB-dependent luciferase reporter gene were purchased from Panomics (RC0002, Freemont, CA, USA). A549 cells were grown in Dulbecco’s modified Eagle’s medium supplemented with 10% foetal calf serum, 100 units/mL penicillin and 100 μg/mL streptomycin. All cells were incubated at 37°C in a humidified atmosphere of 5% CO_2_–95% air.

### Cellular toxicity

For assessing the cellular toxicity of the test fatty acids, 3.0 × 10^5^ mouse splenocytes or purified T-cells, or 5.0 × 10^3^ A549 cells were independently incubated in flat-bottomed microtiter 96-well plates, along with various concentration of nPA dissolved in dimethyl sulfoxide (DMSO) and added as a final DMSO concentration of 0.1%. The control fatty acid was palmitic acid, whose carbon chain is the same length as that of PA. Following incubation for 72 h, an Alamar blue assay was performed according to the manufacturer’s instructions (Invitrogen, Gaithersburg, MD, USA). Fluorescence was measured with excitation at 550 nm and emission at 590 nm using a Varioskan Flash 2.4 (Thermo Fisher Scientific Inc., Waltham, MA, USA).

### IFN-γ secretion

The 1.5 × 10^5^ mouse splenocytes were incubated as described for the cellular toxicity assay. Cells were stimulated with 10 μg/mL phytohaemagglutinin (PHA) for T-cell activation, along with various concentrations of nPA or the control palmitic acid. After incubation at 37 °C for 72 h, culture supernatants were harvested, and IFN-γ secretion was measured by ELISA using a commercially available kit (Biolegend, Inc., San Diego, CA, USA). Similar experiments were conducted using purified T-cells stimulated with 50 ng/mL phorbol 12-myristate 13-acetate (PMA) and 500 ng/mL ionomycin and 48 h incubation.

### Quantitative reverse-transcription polymerase chain reaction (qRT-PCR) for IFN-γ and T-bet mRNA levels

The 2.0 × 10^6^ mouse splenocytes were incubated in flat-bottomed 24-well plates along with test substances, and were stimulated with 10 μg/mL PHA. Following incubation at 37 °C for 24 h, total RNA was extracted from the stimulated splenocytes using TRIzol reagent (Life Technologies, Inc., Grand Island, NY, USA). cDNA was synthesized from 0.5 μg total RNA using ReverTra Ace (Toyobo Co., Ltd., Osaka, Japan). qRT-PCR was done in the AriaMx Realtime PCR system (Agilent Technologies, Inc., Santa Clara, CA, USA) with a commercially available kit (Brilliant III Ultra-Fast SYBR Green QPCR Master Mix, Agilent Technologies, Inc.) as per the manufacturer’s instructions. The expression levels of IFN-γ, T-bet and the housekeeping gene GAPDH were assessed using pre-designed primers for each gene (MA025911, IFN-γ; MA114313, T-bet; MA050371, GAPDH, Takara Bio Inc., Shiga, Japan). A threshold was set in the linear part of the amplification curve, and the number of cycles required to reach the threshold was calculated for each gene. Melting curve analysis was performed to confirm the purity of the amplified bands. Normalization was done using GAPDH mRNA as an internal control for IFN-γ and T-bet mRNA. Similar experiments were conducted using purified T-cells stimulated with PMA and ionomycin as described above.

### NF-κB reporter assay

A549 cells transfected with an NF-κB-dependent luciferase reporter gene were seeded in flat-bottomed 24-well plates at a concentration of 2.5 × 10^4^ cells per well. Cells were stimulated with 20 ng/mL tumor necrosis factor (TNF)-α (Peprotech, Rocky Hill, NJ, USA), following 24 h pre-incubation with test substances. Medium was removed 7 h after addition of TNF-α, and cells were lysed with 50 mM Tris-HCl buffer (pH 8.5) containing 1% Triton X-100. The transcriptional activity of NF-κB was determined by mixing cell lysates with a luciferase substrate (Steady-Glo Luciferase Assay system, Promega, Madison, WI, USA) according to the manufacturer’s instructions. Luminescence was measured using a Varioskan Flash 2.4 (Thermo Fisher Scientific Inc.). Similar experiments were conducted in the presence of a PPARα antagonist GW6471 (1 μM; Cayman Chemicals, Ann Arbor, MI, USA).

### Statistical analysis

Experiments were independently repeated more than three times to confirm reproducibility. Differences between the DMSO control and the test fatty acids were compared using a one-way analysis of variance followed by the Dunnett Multiple Comparison test, or an unpaired t-test. All statistical analyses were performed using the GraphPad Prism version 6.0 (GraphPad Software, San Diego, CA, USA.)

## Results

### IFN-γ production and T-bet expression of mouse splenocytes

**Fig. 1:**
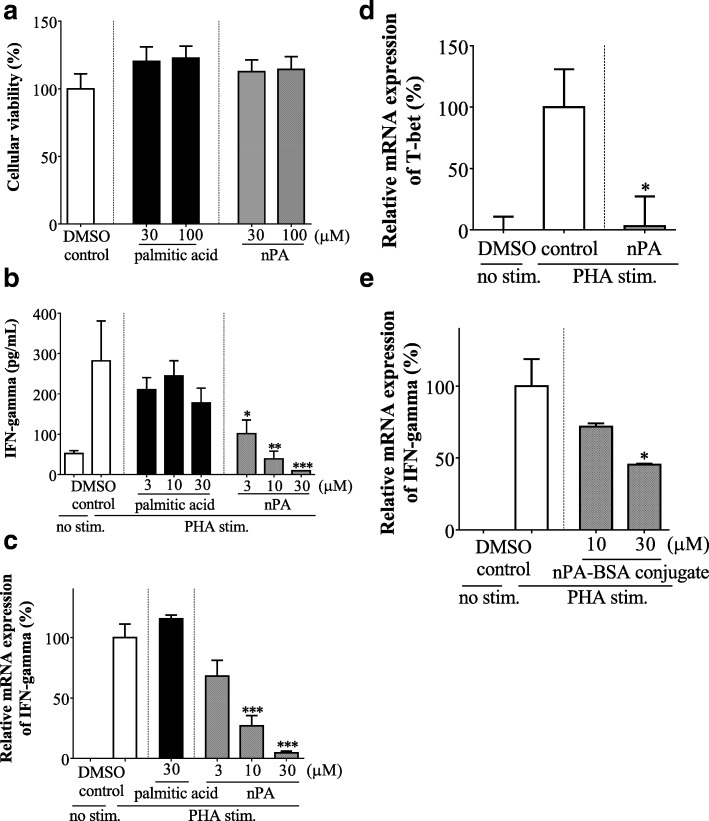
Effects of naturally occurring phytanic acid (nPA) on interferon (IFN)-γ production and T-bet expression in mouse splenocytes. **a** Mouse splenocytes were incubated with palmitic acid or nPA, followed by an Alamar blue assay to determine cell viability. After incubation of phytohaemagglutinin (PHA)-stimulated splenocytes with palmitic acid or nPA, the concentrations of IFN-γ in culture supernatants (**b**), and mRNA expression of IFN-γ (**c**) and T-bet (**d**) were measured by ELISA and qRT-PCR, respectively. **e** Expression of IFN-γ mRNA was determined after PHA-stimulated splenocytes were incubated with nPA-bovine serum albumin (BSA) conjugate. The data represent means ± SEM. *P < 0.05, ** *P* < 0.01 and ***P < 0.001 compared to DMSO (dimethyl sulfoxide) control

To examine the cellular toxicity of test fatty acids, the viability of mouse splenocytes was evaluated after incubation for 72 h. Our results showed that both nPA and the control palmitic acid had no cellular toxicity in concentrations up to 100 μM (Fig. 1a). The effects of nPA on in vitro IFN-*γ* secretion were evaluated after PHA stimulation of mouse splenocytes. nPA reduced IFN-γ secretion in a concentration-dependent manner with near-complete inhibition at 30 μM (Fig. 1b). On the other hand, palmitic acid had no obvious effect on IFN-γ secretion. The inhibitory effects of nPA were also observed at the transcriptional level where mRNA expression of IFN-γ was significantly decreased by treatment with nPA at concentrations higher than 10 μM (Fig. 1c). We also examined the effects of nPA on mRNA expression of the T-box transcription factor T-bet because IFN-γ plays a key role in differentiation of CD4 positive-T-cells to Th1 lineages and T-bet is an important determinant of Th1 cell differentiation. Our results showed that T-bet mRNA expression was strongly suppressed by 30 μM nPA (Fig. 1d). Given that free fatty acids are bound to albumin in human plasma, we also evaluated the immunomodulatory effect of the nPA-BSA conjugate. The results indicated that nPA significantly inhibited IFN-γ mRNA expression even in the form of BSA conjugate (Fig. 1e). Although the inhibitory effect of the nPA-BSA conjugate was slightly less potent than free fatty acids, further studies were carried out using the free form of nPA to simplify experimental procedures.

### T-cell production of IFN-γ

**Fig. 2:**
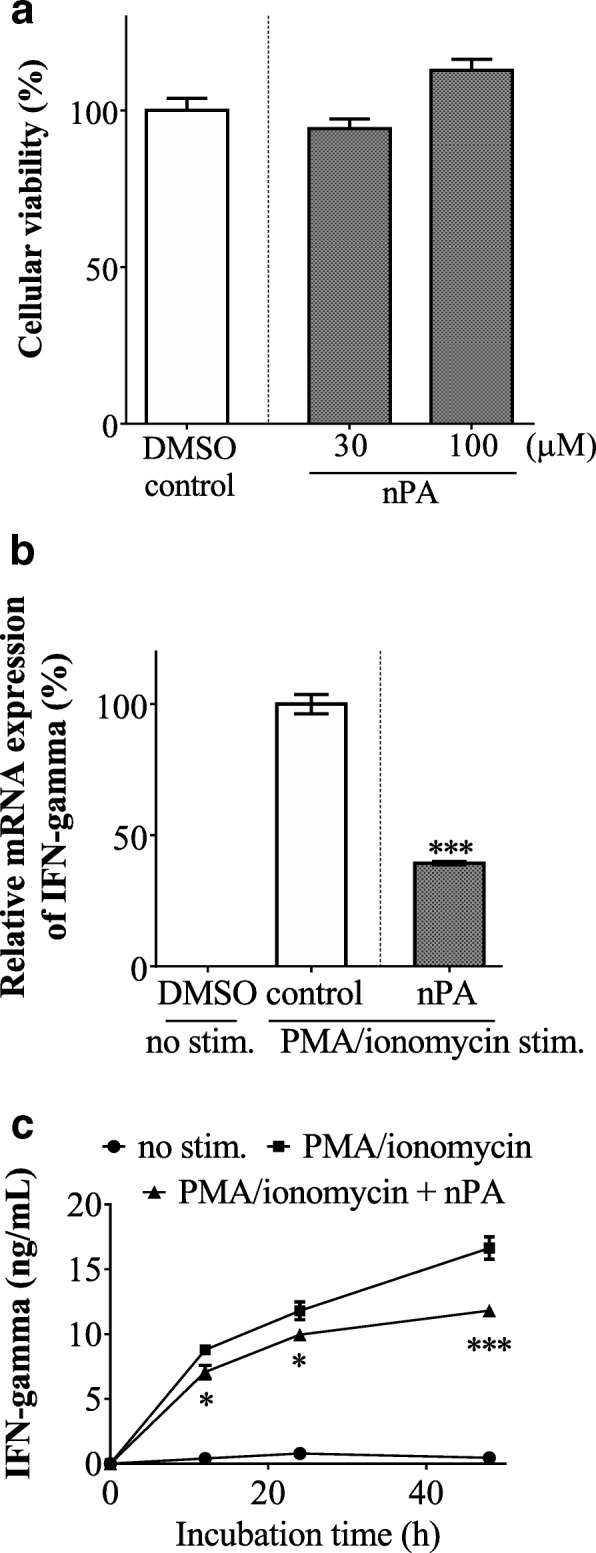
Effects of naturally occurring phytanic acid (nPA) on interferon (IFN)-γ production in purified T-cells. **a** Immunomagnetically purified T-cells were incubated with nPA, followed by an Alamar blue assay to determine cell viability. After T-cells were incubated with nPA under phorbol 12-myristate 13-acetate (PMA)/ionomycin stimulation, IFN-γ mRNA expression (**b**) and concentrations of IFN-γ in culture supernatants (**c**) were measured by qRT-PCR and ELISA, respectively. The data represent means ± SEM. *P < 0.05 and ***P < 0.001 compared to dimethyl sulfoxide (DMSO) control

Splenocytes are composed of variety of leucocytes including T-cells, B-cells, macrophages and dendritic cells, although T-cells are considered the predominant source of IFN-γ production. Therefore, we investigated the direct action of nPA on T-cells. The cellular toxicity of nPA was first evaluated using freshly purified T-cells, showing that nPA had no obvious cellular toxicity in concentrations up to 100 μM (Fig. 2a). The effect of 30 μM nPA on in vitro IFN-*γ* production was evaluated after PMA/ionomycin stimulation of purified T-cells. Our results indicated that nPA significantly inhibited PMA/ionomycin-induced IFN-γ mRNA expression (Fig. 2b). The inhibitory effect of nPA on IFN-γ production was also confirmed at the translational level by evaluation of the time course of IFN-γ secretion after PMA/ionomycin stimulation (Fig. 2c). These findings clearly indicated that nPA elicited its immunomodulatory effects through modification of T-cell function.

### NF-κB reporter assay

**Fig. 3:**
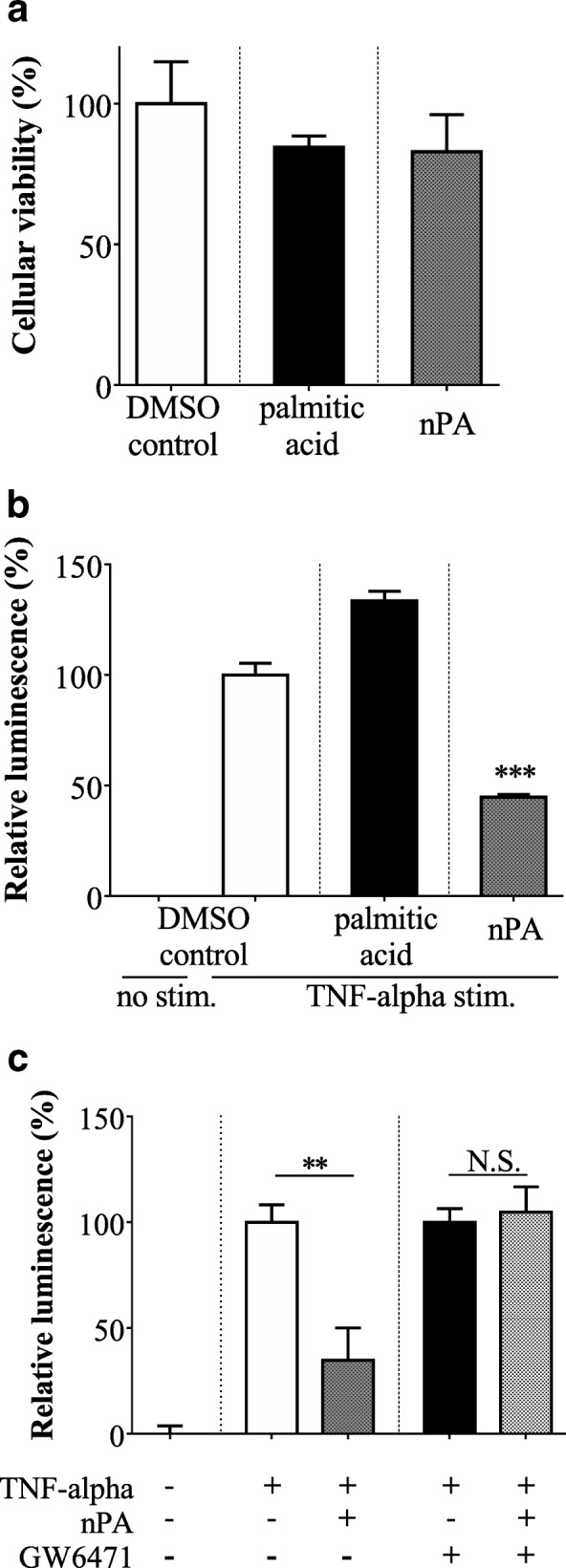
Effects of naturally occurring phytanic acid (nPA) on NF-κB activity in A549 cells. **a** A549 cells with stable expression of an NF-κB-dependent luciferase reporter gene, were incubated with palmitic acid or nPA, followed by an Alamaer blue assay to determine cell viability. **b** After incubation of A549 cells with palmitic acid or nPA, the transcriptional activity of NF-κB was determined by measuring the tumor necrosis factor (TNF)-α-induced luciferase activity. **c** Similar experiments were conducted in the presence of GW6471, an antagonist of peroxisome proliferator activated receptor α (PPARα). The data represent means ± SEM. **P < 0.01 and ***P < 0.001 compared to dimethyl sulfoxide (DMSO) control

To address the potential mechanism for the immunomodulatory effects of nPA, an NF-κB-dependent luciferase reporter assay was employed. No toxicity toward A549 cells was observed with 30 μM of either nPA or the control palmitic acid (Fig. 3a), consistent with the results on mouse splenocytes and purified T-cells. The in vitro effects of 30 μM nPA or palmitic acid on NF-κB-driven transcriptional activity were investigated using A549 cells with stable expression of an NF-κB-luciferase reporter gene. Our results indicated that the control palmitic acid increased rather than decreased NF-κB activity in the A549 cells (Fig. 3b). Contrary to palmitic acid, nPA significantly decreased NF-κB activity (Fig. 3b). The in vitro inhibitory effects of nPA on NF-κB activity were completely abrogated when PPARα was blocked by GW6471 (Fig. 3c), suggesting that PPARα-mediated NF-κB inhibition could be the molecular mechanism for the immunomodulatory effects of nPA.

## Discussion

Unlike straight chain fatty acids which are metabolized by β-oxidation, the metabolism of branched-chain fatty acids proceeds through α-oxidation in the human body. Several reports indicated that the abnormal accumulation of PA in plasma and lipid-containing tissues is one of the clinical signs of adult Refsum disease which is a neurocutaneous syndrome with impaired α-oxidation of branched chain fatty acids [17]. Consequently, the majority of previous studies on PA have focused on its potential toxicity on neuronal cells and its pathogenic role in Refsum disease [18, 19]. Indeed, the plasma concentration of PA in patients with Refsum disease (> 200 μM) proved to be higher than normal (< 30 μM) [17]. In this study, we used a nPA mixture containing equal amounts of the 3*R*, 7*R*, 11*R*- and the 3*S*, 7*R*, 11*R*-isomers for in vitro studies because these isomers are present in the human body. Our results showed no obvious cellular toxicity in mouse splenocytes (Fig. 1a) and purified T-cells (Fig. 2a) when treated with a high dose of 100 μM nPA. Given these findings, we decided to investigate the in vitro immunomodulatory effects of nPA at concentrations up to 30 μM, corresponding to the plasma concentration of healthy humans. This study is the first report of the ability of nPA to inhibit in vitro IFN-γ production by both mouse splenocytes (Fig. 1b and c) and purified T-cells (Fig. 2b and c). In addition, the inhibitory effect of nPA was reproducible in the form of BSA-conjugate which imitates the plasma form (Fig. 1e). These findings suggest that nPA is a promising research target not only as a risk factor for Refsum disease but also as a bioactive food component with immunomodulatory effects.

IFN-γ is the only member of the type II interferons and is well known to be secreted by T-cells as well as by natural killer cells [20]. IFN-γ binds to its ubiquitous receptor on almost all cell types, and consequently IFN-γ signaling provides pleiotropic functions and plays a central role in orchestrating the immune system [21]. In adaptive immunity, IFN-γ production has been considered the hallmark of differentiation of CD4 positive-T-cells into Th1 lineages. T-bet is found in Th1 but not Th2 cells, and is the key transcription factor regulating the development and function of IFN-γ-producing Th1 cells [22]. Our present results showed that the inhibitory effects of nPA on in vitro IFN-γ production were accompanied by decreased levels of T-bet expression (Fig. 1d), implying that nPA could interfere with Th1 cell differentiation. Although IFN-γ is a pivotal cytokine for immunity against bacterial and viral infections, its overexpression and the Th1/Th2 imbalance can lead to the development of IFN-γ-related autoimmune diseases such as multiple sclerosis, inflammatory bowel disease and rheumatoid arthritis [23]. Our findings demonstrate the inhibitory effects of nPA on in vitro IFN-γ production and T-bet expression and suggest that nPA has the potential to attenuate the symptoms of these autoimmune diseases.

Both PA and its metabolite pristanic acid were reported to be natural ligands and activators of several PPAR isoforms, among which PPARα was preferentially activated by nPA [2, 3]. Several studies have demonstrated that PPARα agonists have immunomodulatory effects on T-cells and attenuate the symptoms of autoimmune encephalomyelitis in mouse models of T-cell-mediated disorders [24]. Numerous studies have addressed the molecular mechanism for PPARα-mediated regulation of immune and inflammatory responses, and have found that PPARα activation interferes with NF-κB activity via inhibition of both translocation into the nucleus and transcription initiation [25]. Given that NF-κB is one of the most important transcription factors controlling immune responses (including T-cell production of IFN-γ and Th1 cell differentiation) [26], we hypothesized that nPA elicits inhibitory effects on IFN-γ production through modulation of NF-κB activity. We demonstrated significant inhibitory effects of nPA on the NF-κB-driven transcriptional activity in A549 cells (Fig. 3b). In addition, this study showed that PPARα was required for the inhibitory effects of nPA on NF-κB activity (Fig. 3c). Our studies also found that nPA suppressed in vitro mouse splenocyte production not only of IFN-γ but also of other autoimmunity-associated cytokines such as interleukin-17A (Additional file 1: Figure S1), whose production also requires NF-κB activation [27]. These findings indicate that interference in the NF-κB pathway via PPARα activation is a potential mechanism of the immunomodulatory effects of nPA.

Several researchers have reported variation in the abundance ratio between 3*R*, 7*R*, 11*R*- and 3*S*, 7*R*, 11*R*-PA in the human body according to race, owing to their different ratios in foods [28]. For instance, butter made in New Zealand contains fat with a higher ratio of the 3*R*, 7*R*, 11*R*-isomer, which may correspond to the higher abundance of this isomer in New Zealanders [29]. In this study, we used a nPA mixture containing equal amounts of the 3*R*, 7*R*, 11*R*- and the 3*S*, 7*R*, 11*R*-isomers. Therefore, it cannot be concluded whether one or both isomers are responsible for the immunomodulatory effects. Furthermore, because our present study focused on T-cell production of IFN-γ, the effects of nPA on other types of cells and cytokines have not been determined. Although impaired NF-κB activity is a possible mechanism of the immunomodulatory effects of nPA, it is unknown whether nPA affects the activity of other transcription factors such as NFAT, AP-1 and STAT family members, which also play important roles in immune cell functions. In this study, the immunomodulatory effects of nPA were investigated only in cell-based experiments, and the in vivo effects of nPA have not yet been directly addressed, due to the prohibitively high cost of nPA. The use of cell-based assays, by ourselves and other groups [9, 30], has provided useful insight into the beneficial effects of PA on human health. Further studies are needed to determine the overall effects of nPA on immune responses and to elucidate whether the in vitro immunomodulatory effect of nPA can be reproduced under in vivo physiological conditions.

## Conclusions

In conclusion, the present study demonstrates that nPA inhibits in vitro T-cell production of IFN-γ and that interference in the NF-κB pathway via PPARα activation is a possible mechanism of the immunomodulatory effects of nPA. These findings suggest that nPA is a functional and bioactive fatty acid, and has the potential for amelioration of T-cell mediated autoimmune disease.

## Additional file

Additional file 1: **Figure S1.** Effects of nPA on interleukin-17A production in mouse splenocytes. (DOCX 30 kb)

## Abbreviations

BSA: Bovine serum albumin
DMSO: Dimethyl sulfoxide
IFN: Interferon
nPA: Naturally occurring phytanic acid
PA: Phytanic acid
PHA: Phytohaemagglutinin
PMA: Phorbol 12-myristate 13-acetate
PPAR: Peroxisome proliferator activated receptor
TNF: Tumor necrosis factor
