# Supplementary material for: Naturally occurring 3RS, 7R, 11R-phytanic acid suppresses in vitro T-cell production of interferon-gamma
Source: Lipids Health Dis. 2018 Jun 23;17:147. doi: 10.1186/s12944-018-0793-6 (PMC6015457; doi:10.1186/s12944-018-0793-6)
Supplement: Supplementary file 1 — Figure S1. Effects of nPA on interleukin-17A production in mouse splenocytes. (DOCX 30 kb) [file 12944_2018_793_MOESM1_ESM.docx]

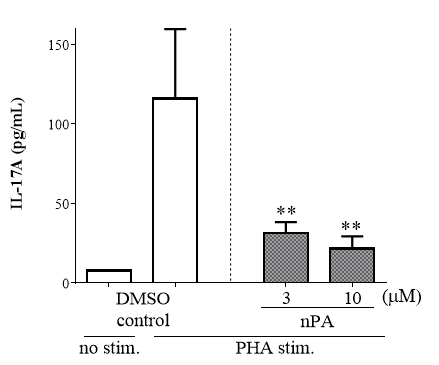


**Figure S1.** Effects of naturally occurring phytanic acid (nPA) on interleukin (IL)-17A production in mouse splenocytes. Phytomaemagglutinin (PHA)-stimulated mouse splenocytes were incubated with nPA, followed by the measurement of concentrations of IL-17A in culture supernatants by ELISA. The data represent means ± SEM. ** P<0.01 compared to dimethyl sulfoxide (DMSO) control.
